# Formal and Informal Care Use Over the Course of Cognitive Deterioration Among Adults With a Disability

**DOI:** 10.1093/geroni/igab046.775

**Published:** 2021-12-17

**Authors:** HwaJung Choi, Kenneth Langa, Edward Norton, Tsai-Chin Choi, Cathleen Connell

**Affiliations:** 1 University of Michigan, Ann Arbor, Michigan, United States; 2 University of Michigan School of Public Health, Ann Arbor, Michigan, United States

## Abstract

The dynamics between formal and informal care among persons with a disability may substantially differ over the course of their cognitive decline.

Based on a nationally representative study of older adults, the analysis sample included 3,685 individuals who had at least one activity of daily living (ADL) limitation. We estimated probabilities of using formal care and informal care in the years before and over the course of dementia after controlling for sociodemographic factors, survey mode, and proxy interview status.

The adjusted probability of receiving care from an informal helper increased before the onset of dementia: 36% in 4 years prior to the onset (T=-4); 46% at T=-2. In contrast, the increase in the probability of using formal care was pronounced primarily at the onset of dementia; for example, the probability of overnight nursing home stay was 12% at T=-2 vs. 31% at T=0, which continued to increase over the subsequent years (39% at T=6). The probability of using nursing home care at the onset was significantly greater for women vs. men (Adjusted risk ratio (ARR)=1.21; p=0.010); non-Hispanic white vs. Hispanic (ARR=1.62; p=0.004); those with low vs. high wealth (ARR=1.60; p < 0.001); those without a spouse vs. with a spouse prior to the onset (ARR=1.39; p < 0.001); and those with all adult children living far vs. at least one coresident adult child prior to the onset (ARR=1.51; p= < 0.001).

Public policies and interventions aimed at providing for the needs of people with dementia should consider disparities in care use across racial/ethnic and socioeconomic groups.

